# Climate change and primary health care in Sahelian Kano, Nigeria

**DOI:** 10.4102/phcfm.v14i1.3745

**Published:** 2022-12-19

**Authors:** Godpower C. Michael, Musa Dankyau

**Affiliations:** 1 Department of Family Medicine, Aminu Kano Teaching Hospital, Kano, Nigeria; 2 Department of Family Medicine, College of Medicine and Heath Sciences, Bingham University Teaching Hospital, Jos, Nigeria

**Keywords:** climate change, primary care, primary health care, Nigeria, Sahel savanna

## Abstract

Climate change (CC) is a global phenomenon that impacts several aspects of human existence. The Sustainable Development Goal 13 implores stakeholders to take action to mitigate the effects of CC. However, its impact on health, particularly primary health care, has not been thoroughly studied. Here, we share anecdotal experiences of the impact of CC on health and primary care presentations in Kano, Northwest, Nigeria. We observed consistent clinical presentations logically associated with high temperatures and excessive flooding in certain months of the year. Presentations range from skin and water-borne diseases to malnutrition and stress-related disorders. Our experience in Kano, Nigeria could be a valuable exposition of the diversity of the impact of CC on primary health care presentation in Africa, considering geographical and cultural differences. It also exposes the paucity of data regarding the impact of CC on primary care activities in Africa.

**Contribution:**

In conclusion, CC has potential impacts on primary health care and practice. The full implications of CC on this vital level of care will require future research (quantitative and qualitative studies). This will help strategic intervention planning by stakeholders.

## Introduction

The years 2010–2019 were regarded as the warmest decade ever, given the increase in global temperature that was accompanied by massive wildfires, hurricanes, droughts, floods and other climate disasters across continents.^1^ By 2015, the United Nations General (UN) Assembly established the 17 sustainable development goals (SDGs), with SDG 13 addressing the need to take urgent action to combat climate change (CC) and its impact.^2^ The targets of this goal were to: (1) strengthen resilience and adaptive capacity to climate-related hazards and natural disasters, (2) integrate CC measures into national policies, strategies and planning, (3) improve education, awareness-raising and human and institutional capacity on CC mitigation, adaptation, impact reduction and early warning, (4) implement the UN Framework Convention in CC and (5) promote mechanisms to raise capacity for planning and management.^2^

Nigeria, a signatory to these conventions, has witnessed noticeable effects of CC. Weather-related disasters have become more frequent. The national adaptation strategy and plan of action on CC for Nigeria reports that the country’s natural and agricultural ecosystems, including freshwater and coastal resources, have been affected by CC.^3^ The direct effects of CC on health result from extreme weather events such as heat waves, floods, droughts, windstorms and wildfires.^3^ On the other hand, the indirect effects of CC on health have been reported to result from malnutrition because of reduced food production, spread of infectious diseases and food- and waterborne illnesses (e.g. typhoid fever and cholera), increased air pollution and high temperatures (correlated with increased cases of meningitis).^3,4,5^

However, vulnerability to the effects of CC varies in different parts of the country. Whereas CC has exacerbated the drought and aridity in northern Nigeria (with the most vulnerable being the northwest and northeast geopolitical zones),^4,6^ southern Nigeria is grappling with changes in the pattern of rainfalls and flooding.^7,8,9^

## Climate change and our experience in Kano, northwest Nigeria

Kano state, with a population of over nine million,^10^ is situated in the northwest geopolitical zone and the Sahel savanna region. Anecdotal reports indicate a pattern of primary care presentations probably dictated by CC. Unpublished clinic records indicate that over the last few years, waterborne diseases such as cholera and other diarrheal diseases peak during April and May when the daytime temperatures could be as high as 41 °C. This could be linked to the dramatic drying up of popular water sources such as boreholes and wells, resulting in a scarcity of potable water.

Moreover, reduction in water bodies such as rivers reduces hydroelectric power generation, leading to inadequate electricity supply and inability to power fans and air conditioners in homes; this leads to increased sweating, and consequently, more patients with heat rashes present at the primary care clinic. Many adults present with fatigue, malaise and daytime somnolence because of poor sleep at night caused by the high temperatures, which can affect workplace productivity. Incidences of deaths of families because of carbon monoxide fumes (poisoning) from small electricity generator sets (used in powering fans) are commonly reported in the media during these periods.^11^

On the other hand, in July and August (the peak of the rainy season), flooding increases. The media was awash in 2021 with reports of severe flooding in parts of Kano state and north-eastern Nigeria, with farms and cattle destroyed by flooding resulting from heavy and prolonged rainfalls. The ripple effect was a poor harvest, high cost of food items and malnutrition (a common presentation in primary care).^3^ In addition, the Boko Haram insurgency, in north-eastern towns and villages, coupled with drought, has led to population migration to cities like Kano.^12^ This has led to noticeable congestion (overcrowding), increased vehicular traffic, air pollution, increased demand for goods and services with the consequent high costs and increased poverty. Related health disorders include mental stress and illnesses,^13^ increased cardiovascular events,^14^ air-borne diseases and malnutrition. Thus, it is not surprising that we now encounter more patients with anxiety disorders, depression, hypertension and upper respiratory infections in primary care, where most citizens interface with the health care system, given the link between these diseases and CC.^15,16,17,18^ Unfortunately, despite the increasing prevalence of these diseases, less than 5% of Nigerians have health insurance coverage.^19^ The uninsured population in the context of global inflation have difficulty affording orthodox medical care; and hence, uptake of unorthodox care (often cheaper with doubtful efficacy) is on the rise. A popular scenario in Kano city is where an unorthodox (traditional) practitioner established his post opposite the gate of a foremost public tertiary hospital, with a high volume of clients.

## Conclusion

The potential impacts of CC on primary health care in Africa are not thoroughly studied and understood. Our primary care experiences in Sahelian Kano, Nigeria, may indicate that future research is required to monitor climate-sensitive conditions and highlight the impact at this important level of care. This could include CC awareness and knowledge studies in practitioner and patient populations.^20^ This will help the stakeholders with information necessary for the strategic planning of appropriate interventions.
